# Mental health and its association with injury risk in elite adolescent athletes- a prospective cohort study

**DOI:** 10.1186/s13102-026-01558-3

**Published:** 2026-02-10

**Authors:** Ida Lindman, Esmaeil Mohammadzadijaran, Adad Baranto, Josefin Abrahamson

**Affiliations:** 1 General Practice/Family Medicine, School of Public Health and Community Medicine, Institute of Medicine, Sahlgrenska Academy, University of Gothenburg, Gothenburg, Sweden; 2 Research, Education, Development & Innovation, Primary Health Care, Region Västra Götaland, Gothenburg, Sweden; 3 Department of Orthopaedics, Institute of Clinical Sciences at Sahlgrenska Academy, University of Gothenburg and Sahlgrenska University Hospital, Gothenburg, Sweden; 4 Orthopaedic Research Unit, Sahlgrenska University Hospital, Gothenburg, Sweden; 5 Department of Health and Rehabilitation, Institute of Neuroscience and Physiology at Sahlgrenska Academy, University of Gothenburg, Gothenburg, Sweden

**Keywords:** Athletes, Adolescent, Mental health, Sports/injuries, Youth sports

## Abstract

**Background:**

Adolescent athletes face an increased risk of both sports-related injuries and mental health challenges. With the rising prevalence of these issues, understanding their interaction is increasingly important. This study aimed to evaluate the mental health of adolescent athletes, examining differences by sex and type of sport. Additionally, it aimed to investigate the association between mental health and sports-related injuries.

**Methods:**

A prospective study including elite adolescent athletes between 16–19 years old. Mental health was assessed by the short version of the Warwick-Edinburgh Mental Wellbeing Scale (SWEMWBS) to capture mental wellbeing and one additional question assessing perception of poor mental health. Prospective injury was defined as any self-reported physical complaint by the athlete. Logistic regression analysis was performed to study the influence of mental health variables as potential risk factors for injuries.

**Results:**

A total of 171 athletes were included (16–19 years), where 48% reported having had a severe injury past year. Mental health issues were significantly more prevalent among females (36%), who also had a significantly lower mean SWEMWBS score compared with males. Female ice hockey players reported significantly higher rates of self-reported periods of poor mental health (*p* = 0.03) and lower SWEMWBS scores compared to their male counterparts (*p* < 0.001). Athletes who reported a new injury during the follow-up had significantly lower SWEMWBS score compared with injury-free athletes (*p* = 0.01). No other association was found between injuries and mental illness or the SWEMWBS score.

**Conclusion:**

This study found that one-fourth of adolescent athletes reported at least one period of poor mental health, with a significant overrepresentation of female athletes. Notably, female ice hockey exhibited both higher rates of poor mental health and lower scores of mental wellbeing compared with their male counterparts. Regarding injuries, lower SWEMWBS score was found among athletes who reported new injuries during the follow-up. These findings highlight the need for increased awareness and targeted mental health support within youth sports, particularly for female athletes. Future research should further investigate the underlying causes of these disparities and explore effective prevention and intervention strategies to promote mental wellbeing among adolescent athletes.

**Supplementary Information:**

The online version contains supplementary material available at 10.1186/s13102-026-01558-3.

## Introduction

Adolescence is a critical period of physical, emotional, and psychological development, marked by increased vulnerability to both mental health challenges and physical injury [1, 2]. Among adolescent athletes, the concomitant pressures of competitive sports, academic demands, and social expectations can often exacerbate mental health concerns, potentially influencing their overall wellbeing. Mental health problems such as anxiety, depression, and stress have been shown to be as prevalent among athletes as the general population [3]. However, some evidence suggests that athletes might be at greater risk than non-athletes and there is a concern that the prevalence is underreported [4–6]. While physical activity is well-known to increase mental health [7, 8], the demands including intrinsic and extrinsic stressors on elite athletes may instead increase their susceptibility to poorer mental health [3, 6]. Additionally, being in the adolescent stage of development further amplifies their vulnerability to mental health challenges [2]. However adolescent athletes are not as studied as their senior counterparts.

In recent years, there has been growing recognition of the importance of mental health in the context of sports [9]. It is well known that mental health problems can negatively impact an athlete's ability to perform [3, 7, 10]. Yet, the relationship between these psychological factors and the risk of physical injury remains underexplored. Injuries can have significant short- and long-term effects on adolescents' physical health and emotional wellbeing. Likewise, recent evidence has suggested that athletes with poor mental health may be at higher risk of both injury and prolonged recovery time [1, 11]. While mental health and injury thus creates a complex cycle, the specific mechanisms linking mental health to injury risk in adolescent athletes are not fully understood.

Given the increasing prevalence of both mental health issues and sports-related injuries among adolescents, it is crucial to better understand how these factors interact. This knowledge could lead to more effective prevention strategies, identification of at-risk athletes, earlier interventions, and improved support systems for young athletes. It has been concluded that more high-quality epidemiological studies are warranted to identify mental health among athletes, especially adolescents [3].

The primary aim of this study was to evaluate the mental health of adolescent athletes and examine differences between sex and sports type. The secondary aim was to assess how mental health was associated with sports-related injuries.

## Methods

This is a prospective cohort study. The study is part of the Healthy Injury-Free Adolescent Athletes Project (HIFAA) [12].

### Ethics approval and consent to participate

The HIFAA project including this study has ethical approval from the Swedish Ethical Review Authority with the diary number: 2021–05496-01**.** Prior to participation, athletes were provided with both oral and written information about the study and gave written informed consent. All responses were kept confidential, stored in password-protected files, and accessible only to the research team. Participation was voluntary, and athletes could withdraw at any time without consequence. No participant was under the age of 16 years.

### Study population

Eligible participants were 1) current students at Swedish high schools (age 16–19 years old), and 2) athletes actively training and competing in a sport with the goal of reaching a national or international competitive level. Athletes participating at a recreational level were excluded.

High schools were selected based on their association with local sports clubs, national federations, and personal contacts, ensuring a diverse representation from both urban and suburban areas across Sweden.

### Data collection

Participants completed a baseline questionnaire at the start of the study. Each week, they received an automatic text-message with a personal link to the weekly questionnaire. Supplementary Material 1 shows the questionnaires. Non-responders were sent a reminder the following day. Both questionnaires were digital and linked to a web application developed for the HIFAA project. Data collection took place from December 2022 to December 2023.

The baseline questionnaire included data on demographics, weekly training and competition/match play frequency, and a history of severe injuries within the past year. A severe injury was defined as one that caused the athlete to not being able to fully participate in regular training and/or competition for at least one month [13].

Mental health was assessed by two measures. Firstly, the short version of the Warwick-Edinburgh Mental Wellbeing Scale (SWEMWBS) to capture mental wellbeing [14, 15]. The SWEMWBS is a validated instrument assessing wellbeing which has been translated and validated in Swedish, and is widely used in national surveys of adolescents [14, 15] Lower scores indicate poorer mental wellbeing. It demonstrates good reliability (Cronbach's alpha 0.86–0.88) and strong face and criterion validity [14]. Secondly, to assess the perception of having a history of poor mental health, an additional question was also included: "Have you ever experienced a period of poor mental health (lasting at least two weeks daily) that significantly impacted your daily life and sports performance?" (yes/no) [16]. The item was developed by an interdisciplinary expert group (physician, physiotherapist, psychologist) and piloted among adolescent athletes in the target population, ensuring strong face validity.

Data addressing injuries were self-reported through the weekly questionnaire by questions from the OSTRC-H (The Oslo Sport Trauma Research Center Questionnaire on Health Problems) [17]. An injury was classified as any physical complaint sustained by an athlete that resulted from a match play/competing or training, irrespective of the need for medical attention or time loss from activities [18].

### Data analysis

Descriptive statistics are presented as mean and standard deviation (SD) for continuous variables and as frequency with proportion (%) for categorical data. Chi^2^ tests were used for comparisons between categorical data and independent t-tests for continuous data. The normal distribution of data was assessed using histograms. A binary logistic regression analysis was performed to study the influence of mental wellbeing variables as potential risk factors for injuries. To be included in this analysis, athletes had to be injury-free for at least four consecutive weeks and answered at least 10% of all weekly questionnaire [19, 20]. Injured athletes at study start (*n* = 69, 47%) were followed until they had more than four consecutive weeks of reporting full participation in normal training and/or had a new injury not related to the initial injury [20]. Throughout calculations, the significance level was set at *p* < 0.05. All statistical analyses were performed using IBM SPSS Statistics (Version 30.0 for Mac).

## Results

A total of 171 (104 males and 67 females) athletes answered the baseline questionnaire, which corresponds to 100% of the athletes asked to participate in the study. Mean age was 16.9 (SD 0.8) years with no sex difference. A total of 50% and 45% reported having had a severe injury past year, in male and female athletes respectively. Table 1 shows demographics stratified by sex.

**Table 1:** Demographics stratified by sex

	**Males**	**Females**	**Total**
Participants, *n* (%)	104 (60.8%)	67 (39.2%)	171 (100%)
Age, years, mean (SD)	17.0 (0.8)	16.8 (0.8)	16.9 (0.8)
Sport, *n* (%)			
Ice hockey	33 (63.5%)	19 (36.5%)	52 (100%)
Soccer	30 (68.2%)	14 (31.8%)	44 (100%)
Track & Field	15 (44.1%)	19 (55.9%)	34 (100%)
Bandy	15 (88.2%)	2 (11.8%)	17 (100%)
Gymnastics	2 (22.2%)	7 (77.8%)	9 (100%)
Floorball	3 (37.5%)	5 (62.5%)	8 (100%)
Fencing	6 (100%)	0 (0%)	6 (100%)
Tennis	0 (0%)	1 (100%)	1 (100%)
Severe injury past year, *n* (%)	52 (50%)	30 (44.5%)	82 (48%)
Injury at study start, *n* (%)	41 (48.8%)	28 (45.2%)	69 (47.3%)^†^
New injury, *n* (%)	65 (77.4%)	53 (85.5%)	118 (80.8%)^†^
Period of poor mental health, *n* (%)	20 (19.2%)	24 (35.8%)*	44 (25.7%)
SWEMWBS, mean (SD)	25.6 (3.7)	23.2 (3.6)**	24.6 (3.8)

*n* Numbers, *SD* Standard deviation, *SWEMWBS* the short version of the Warwick-Edinburgh Mental Wellbeing Scale

^†^Missing values: 25 (20 male; 5 female)

*Chi2-test, *p* = 0.015 between the sex

**Independent t-test, *p* < 0.001 between the sex

Athletes who reported having had a period of poor mental health had a significantly lower SWEMWBS score (mean 22.1, SD 3.7) compared with those not having had a period of mental illness (mean 25.5, SD 3.5 [*p* < 0.001]).

A significant higher proportion of female athletes reported having had a period of poor mental health (36%) and had a significantly lower mean SWEMWBS-score (23.2, SD 3.6) compared with male athletes in total (Table 1).

**Fig. 1:**
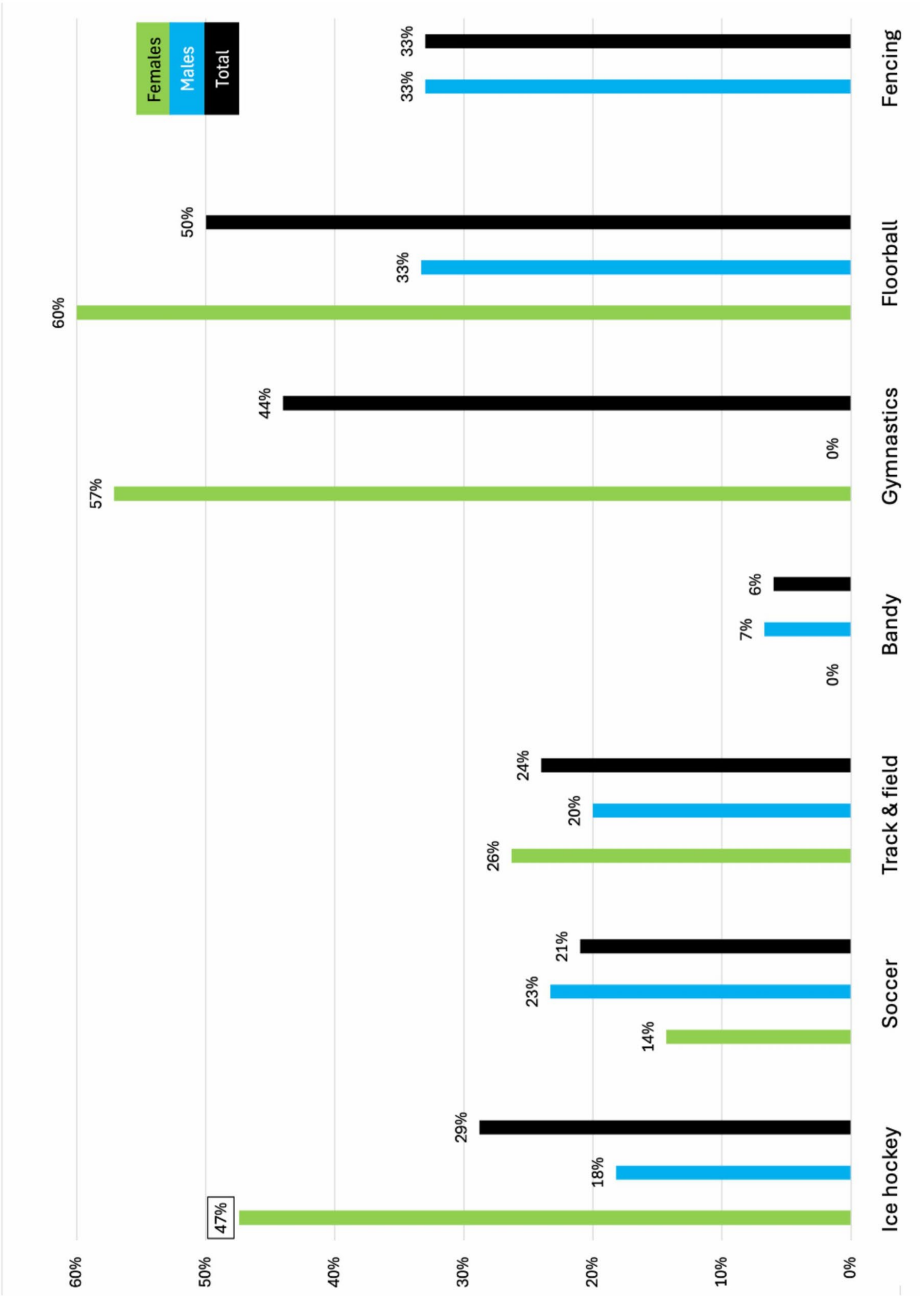
Proportion of athletes reporting having had a period of poor mental health stratified by sport and sex. Squared number indicates a significant difference between the sex

**Fig. 2:**
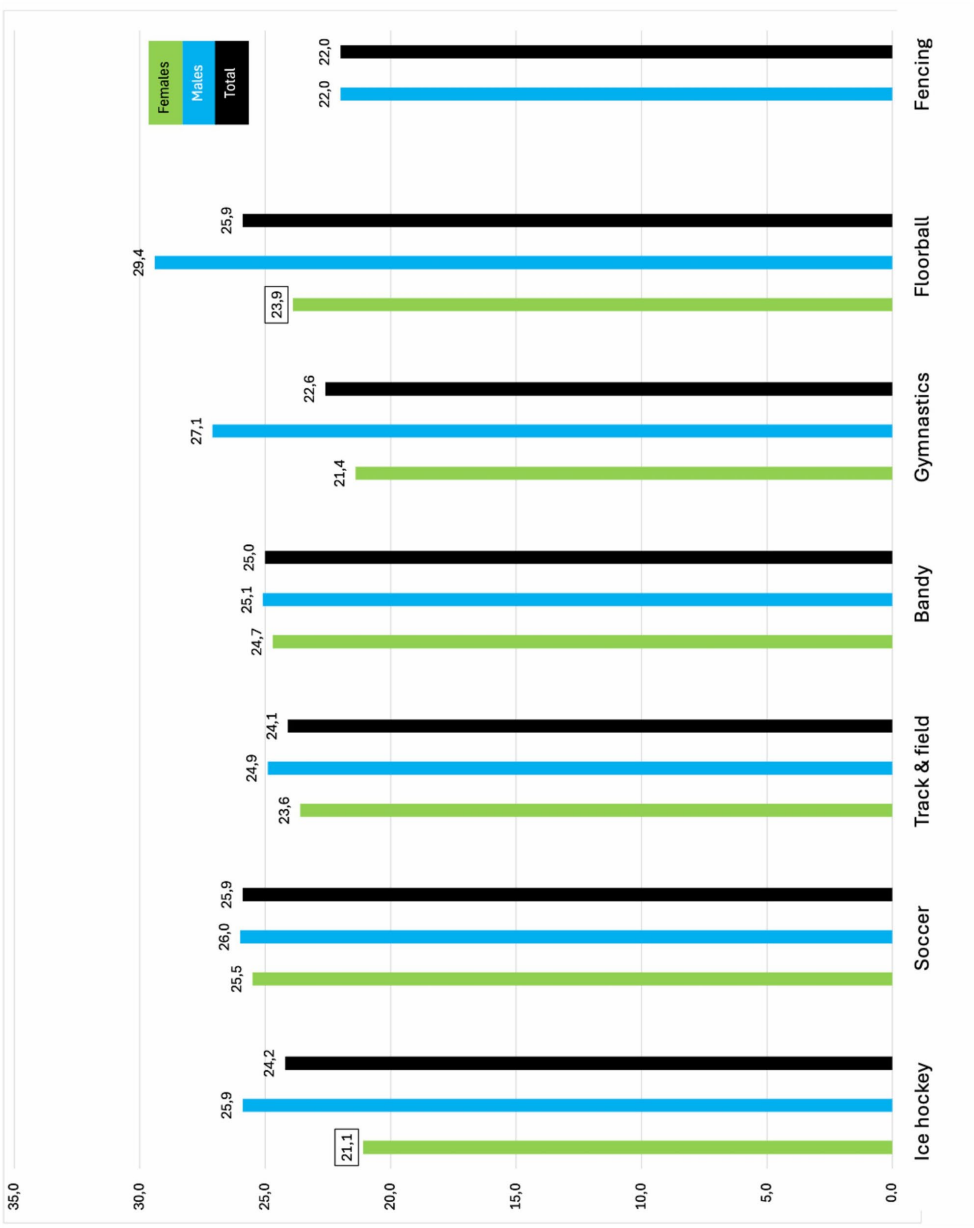
Mean SWEMWBS (the short version of the Warwick-Edinburg Mental Wellbeing Scale) stratified by sport and sex. Squared number indicates a significant difference between the sex

Figures 1 and 2 show the proportion of athletes reporting having had a period of poor mental health and mean SWEMWBS score, respectively, in total and stratified by sex. There was no significant difference regarding type of sport, however, there was a significant sex difference in ice hockey for both having had a period of poor mental health (*p* = 0.03)(Fig. 1) and SWEMWBS score (*p* < 0.001)(Fig. 2).

### Injuries versus mental health

In total, there were 25 (14.6%) dropouts (ie, responed only to the baseline questionnaire), leaving 146 athletes for the comparisions between injuries and mental health. The Chi2 analysis showed no association between having had a severe injury (respons rate 100% with dropouts), being injured at study start (respons rate 100% without dropouts) or getting a new injury during the follow-up (mean response rate 47% without dropouts), and reporting having had a period of poor mental health.

The independent t-test revealed that athletes who reported a new injury during the follow up had significantly lower SWEMWBS score (mean 24.3, SD 3.6) compared with those who were injury-free (mean 25.9, SD 3.8 [*p* = 0.01])(mean response rate 47%). No other associations were found between the SWEMWBS score and injuries.

Table 2 shows the multivariable logistic regression analysis of risk factors for injury in total. A total of 118 (69.0%) athletes were included in the binary regression analysis (25 dropouts [14.6%] and 28 [16.4%] athletes were injured at study start and did not complete at least four consecutive weeks of full training during the follow-up period). Mean weekly respons rate was 52%. The regression analysis did not reveal any significant factors for having a new injury during the follow up.

**Table 2:** Multivariable logistic regression analysis of risk factors to a new injury within 1 year

Variabel	OR (95% CI)	*P*-value
SWEMWBS	0.904 (0.769–1.063)	0.22
Period of mental illness *(reference: yes)*	1.930 (0.364–10.239)	0.44
Sex *(reference: female)*	1.727 (0.575–5.184)	0.33
Age	0.685 (0.351–1.336)	0.27
Severe injury past year *(reference: yes)*	2.343 (0.821–6.691)	0.10

*OR* Odds Ratio, *CI* Confidence interval, *SWEMWBS* the short version of the Warwick-Edinburgh Mental Wellbeing Scale

## Discussion

The most important finding of this study was that one out of four (25.7%) of the adolescent athletes reported having had a period of poor mental health. Furthermore, a significantly higher proportion of female athletes reported having had a period of poor mental health compared with their male peers (36% vs. 19%). This was especially noted in ice hockey female players where almost every second reported having had a period of poor mental health.

The secondary aim of this study was to prospectively evaluate the association between mental health and sports injuries. Despite increasing recognition of the interplay between mental health and injury risk, prospective studies on this topic remain sparce [21]. Previous research has suggested a complex, bidirectional association, whereby poor mental wellbeing may increase the risk of injury, and injuries may, in turn, contribute to the onset or exacerbation of mental health problems [21]. Several studies have reported associations between poor mental wellbeing and subsequent injury risk [1, 11, 22–24], while others indicate that injury can negatively affect mental health [25, 26]. In this study, 81% of participants reported a new injury during the follow up. However, only one statistically significant association was found between injuries and mental health. Specifically, athletes who sustained a new injury reported significantly lower SWEMWBS score compared with those who remained injury-free during the follow-up. Although the difference of 1.6 points on the SWEMWBS was statistically significant, its clinical relevance may be argued. However, a previous study, though not limited to athletes, suggested that a difference of 1 point may represent a meaningful difference at the individual level and can be used as a minimal clinically important difference (MCID) [27]. Furthermore, much of the existing literature suggesting a link between mental health and injury has primarily focused on specific psychiatric diagnoses, symptom profiles, or specific sports, which may limit comparability [11, 22, 24, 28, 29]. For example, athletes with anxiety and depression have been reported to be at a higher risk of injury, even after adjusting for prior injuries [22]. Several factors may explain the absence of a significant relationship in this study. Differences in study design, population characteristics, and mental health assessment tools could contribute to the discrepancy between the results and those of previous research. Despite this, the study adds valuable prospective data to a field where such research remains limited.

### Mental health in general

A key finding of this study was the high prevalence of at least one period of poor mental health among adolescent athletes. It has been suggested that athletes experience a similar risk of poor mental health as to the general population [3, 30]. However, some evidence indicate that elite athletes may have an even higher prevalence of poor mental health [31]. This study aligns with this research, confirming that poor mental health is a significant concern in this population. While this study did not differentiate between type of poor mental health, previous research has shown risk of conditions such as anxiety, depression, eating disorders, stress-related disorders and substance use, including alcohol misuse, among athletes [30, 32]. Moving forward, it is crucial for future studies to explore the underlying causes of poor mental health in athletes, particularly adolescents, to develop effective prevention strategies and interventions aimed at improving mental health within this group.

### Sex differences in mental health

A significant difference in mental health outcomes was observed between male and female athletes, with female athletes reporting higher rates of poor mental health and lower scores on the SWEMWBS. These findings align with previous research indicating that female athletes are at a greater risk of experiencing mental health issues compared with their male counterparts [5, 30, 33]. Studies have consistently shown that female athletes report higher levels of anxiety, depression, and psychological distress, a trend that mirrors patterns observed in the general population [30]. This aligns with previous research showing higher prevalence of mental health concerns among female athletes. While several factors may contribute to this disparity, one possibility is that male athletes underreport psychological distress due to stigma, particularly in male-dominated sports cultures. Such reporting biases may partly explain the observed sex differences and should be considered when interpreting results [5, 34]. Future research should aim to identify both sport-specific and societal risk factors contributing to sex differences in mental health. Longitudinal studies are particularly needed to track how symptoms develop and persist over time. In parallel, sports organizations should consider implementing preventive strategies, promoting mental health awareness, and creating supportive environments tailored to the unique needs of female athletes.

### Mental health in sports

This study examined mental health in relation to both sex and sport type. The findings revealed that female ice hockey players reported lower rates of mental health compared with their male peers, and athletes in other sports. Female ice hockey remains an understudied population, and although the sport has grown in popularity in recent years, the high prevalence of poor mental health among these athletes is concerning. Nearly half of the female ice hockey players in this study reported experiencing at least one period of poor mental health, along with significantly lower wellbeing scores on the SWEMWBS, indicating poorer mental health.

Several factors could contribute to these findings. Female athletes, particularly those in male-dominated or physically demanding sports like ice hockey, may face unique stressors, including societal expectations, performance pressure, limited resources, and sex-related barriers within their sport. The psychological demands of competing in environments traditionally dominated by men, as well as potential disparities in coaching support and access to mental health resources, may also play a role in the higher prevalence of poor mental health observed in these athletes. Further research is needed to explore these contributing factors and to develop targeted mental health interventions for female athletes in these sports.

### Strengths and limitations

The prospective study design along with the inclusion of a diverse range of sports enhances the generalizability of the findings. Furthermore, the inclusion of both self-reported mental illness as well as the validated and well-established questionnaire SWEMWBS strengthens the study by providing a standardized measure of mental health.

However, there are also important limitations to consider. First, the study included a relatively small sample size (171 participants), which may limit the statistical power and increase the risk of a type II error, particularly when analyzing differences between specific sports. Although a variety of sports were represented, some had only a small number of participants, making it difficult to draw firm conclusions about sport-specific trends.

Second, all data were self-reported, which introduces the possibility of response bias. Self-reported injury data may be subject to recall bias or misclassification, and the reporting of mental health may be influenced by stigma, particularly among male athletes. As discussed, underreporting of mental health concerns in male athletes could contribute to the observed sex differences and may mask the true prevalence of self-reported poor mental health in this population. Future studies should consider incorporating clinical assessments or alternative measures to mitigate this risk.

Furthermore, the study did not include detailed diagnostic criteria or symptom-based assessments of mental health. While self-reported experiences provide valuable insights, the lack of clinical diagnoses or standardized symptom screening tools limits the ability to distinguish between different types and severities of mental health conditions. The SWEMWBS provided a validated and robust measure of mental wellbeing. In contrast, the single-item question on previous poor mental health, although developed by an expert group and piloted with adolescent athletes to ensure strong face validity, is not a fully validated measure. Findings based on this item should therefore be interpreted with caution. Future research should aim to integrate both self-reported data and objective clinical measures to improve accuracy and depth of understanding.

Despite these limitations, this study contributes to the growing body of literature on mental health in adolescent athletes and highlights the need for further research with larger sample sizes, objective diagnostic measures, and longitudinal designs to better understand the complex relationship between mental health and sports participation.

### Conclusion

This study found that one-fourth of adolescent athletes reported experiencing at least one period of poor mental health, with a significant overrepresentation of female athletes. Notably, female ice hockey players exhibited higher rates of poor mental health compared to their male counterparts, along with lower wellbeing scores. Regarding injuries, lower SWEMWBS score was found among athletes who reported new injuries during the follow-up. These findings highlight the need for increased awareness and targeted mental health support within youth sports, particularly for female athletes. Future research should further investigate the underlying causes of these disparities and explore effective prevention and intervention strategies to promote mental wellbeing among adolescent athletes.

## Supplementary Information

Supplementary Material 1.

## Data Availability

The datasets analysed during this project will not be publicly available due to ethical restrictions. However, it will be available from the authors on reasonable request.

## Abbreviations

SWEMWBS: The short version of the Warwick-Edinburgh Mental Wellbeing Scale
HIFAA: The Healthy Injury-Free Adolescent Athletes Project
OSTRC-H: The Oslo Sport Trauma Research Center Questionnaire on Health Problems
SD: Standard deviation
n: Number
