# Supplementary material for: Mental health and its association with injury risk in elite adolescent athletes- a prospective cohort study
Source: BMC Sports Sci Med Rehabil. 2026 Feb 10;18:131. doi: 10.1186/s13102-026-01558-3 (PMC12990549; doi:10.1186/s13102-026-01558-3)
Supplement: Supplementary file 1 — Supplementary Material 1. [file 13102_2026_1558_MOESM1_ESM.docx]

**Supplementary material 1 – The Baseline questionnaire and the weekly questionnaire**

**NOTE!** This is a direct translation of the consent form, baseline and weekly questionnaires, from Swedish to English. The athletes received the form in Swedish, and the English translation has therefore not been validated in any way.

**BASELINE QUESTIONNAIRE including consent form**

**Research Project: Injuries, physical and psychological load among young athletes**

This project aims to investigate the occurrence of injuries, physical and psychological load, and how these factors are associated in young athletes.

We received your name from your coach/teacher/parent and are now contacting you to ask whether you are interested in participating in this research project regarding sports injuries and load.

The aim of the project is to study injuries and its association with physical and psychological load among young athletes. We aim to track injuries and load over a longer period (up to 3 years), and thereafter conduct a 5-year follow-up after graduation from high school. We hope this research will increase the knowledge of causes to sports injuries and provide better ways to prevent them.

The participation in this project includes answering a number of questions about your sport background, previous injuries, and mental health. These questions will also be answered annually. In addition to this, you will answer a small set of questions every week regarding injuries, pain and load. All questions will be answered via a web app linked to your smartphone, where you also will be able to view some of your own results from the past months. You will follow until graduation and thereafter receive some follow-up questions 5 years after your graduation.

Participation in this project is totally voluntary, and you may withdraw at any time. Your information, answers, and results will be treated confidentially, and no unauthorized persons will have access to the data. Results will be presented at group level, and no individual can be identified. Your answers will not affect your chances getting into teams or competitions, and will **not** be shared with coaches, teachers or parents. The results will be compiled and used in studies on injuries, load and sports. The research is conducted under the responsibility of the Västra Götaland Region. The study has been reviewed and approved by the Swedish Ethical Review Authority.

The data will be stored according to the EU General Data Protection Regulation (GDPR), securely locked for at least 10 years to allow for retrospective review. The controller for your personal data is Sahlgrenska University Hospital, and the data is handled in accordance with the GDPR (2018:218). You have the right to access your personal data free of charge and request corrections if necessary. You can also request your data to be deleted or that its processing being restricted. To access your data, contact the Data Protection Officer at Sahlgrenska University Hospital by phone at +46 (0)31-3432715 or by email at sahlgrenska.universitetssjukhuset.dso@vgregion.se. If you are dissatisfied with how your data is processed, you have the right to lodge a complaint with the Swedish Authority for Privacy Protection (IMY).

This project is not covered by any insurance. No compensation is provided. If you would like to receive a summary of the results and/or have questions about the study, you are welcome to contact the study leaders.

## Contact Information for Study Leaders

Josefin Abrahamson, PhD, Physiotherapist, Research Unit Orthopedics, Sahlgrenska University Hospital/Mölndal Hospital, josefin.abrahamson@vgregion.se

Ida Lindman, PhD, General Practitioner, Sahlgrenska Academy, University of Gothenburg, ida.lindman@gu.se

## Consent

In order to use your answers anonymously in our research, we need your consent:

☐ I consent to my answers being used in the research

1. First and last name_________________________________________________
2. Birth date ________________________________________
3. Phone number ________________________________________________
4. E-mail __________________________________________________
5. Have you moved from home?

☐ Yes

☐ No *(go to question 7)*

1. How old were you when you moved from home? ________________years
2. Before high school, did you attend a school with sports profile/specialization?

☐ Yes

☐ No

1. Are you attending any sports high school?

☐ Yes, I attend a National sprt high school (RiG)

☐ Yes, I attend a National Sports Education program (NiU)

☐ Yes, I attend a Local Sports Education program (LiU)

☐ No *(go to question 10)*

1. How important was it for you to attend a sports high school?

☐ Very important

☐ Important

☐ Moderat important

☐ Slightly important

☐ Not important

1. Did you train extra to increase your chances of getting into a sports high school?

☐ Yes

☐ No

1. What school grade are you in?

☐ 1

☐ 2

☐ 3

☐ 4

# YOUR SPORT

1. What is your main sport? __________________________________________
2. Are you active in other competitive sports beside your main sport?

☐ Yes, namely _________________________________

☐ No

1. How old were you when you seriously started to, during the entire year, focusing on your main sport (i.e. began intensive, year-round training and excluding other sports)?

_______________ yeas

1. My main sport is:

☐ Team sport

☐ Individual sport

1. What position do you have in your team?

☐ Field player

☐ Goalkeeper

1. I train and play matches with:

☐ Juniors

☐ Seniors

☐ Both juniors and seniors

1. On average, how many days in the week are you training? *(All kind of training: sport specific, strength, cardio, schoolsport)*

☐ 0

☐ 1

☐ 2

☐ 3

☐ 4

☐ 5

☐ 6

☐ 7

1. Including all sessions per week (also school sports), how many sessions do you train on average per week?__________________
2. **Teamsport**: **On average, how many matches do you play per week?** *(during match and pre-season)*

☐ 0.5

☐ 1

☐ 1.5

☐ 2

☐ 2.5

☐ 3

☐ 4

☐ Fler än 4

1. **Individual sport: On average, how many competitions per month do you participate in?** *(during competition season)* ___________________ competitions

### QUESTIONS ABOUT THE PAST YEAR

1. **Have you had a significant/serious injury in the past YEAR?** Namely, an injury that prevented you from participating in your regular training/competition/matches for at least 1 month
   ☐ Yes, list all injuries if more than one_______________________________________

______________________________________________________________________

☐ No *(go to question 24)*

1. Have you been examined by a physician and/or physiotherapist for your injury/injuries?

☐ Yes

☐ No

1. Have you had an MRI, X-ray, or other examination for your injury/injuries?

☐ Yes

☐ No

1. Have you received a diagnosis for your injury/injuries?

☐ Yes, namely *(list all if you had more than one)* ______________________________

☐ No

1. **How have your training habits changed over the past year?**
   ☐ Increased significantly
   ☐ Increased
   ☐ No change
   ☐ Decreased
   ☐ Decreased significantly
2. **How have your competition/match habits changed over the past year?**☐ Increased significantly
   ☐ Increased
   ☐ No change
   ☐ Decreased
   ☐ Decreased significantly
3. **How have your sleep habits changed over the past year?**☐ Sleeping much more (go to question 29)
   ☐ Sleeping more (go to question 29)
   ☐ No change (go to question 29)
   ☐ Sleeping less
   ☐ Sleeping much less
4. **Why have you reduced your sleep?** _________________________________________
5. **How many hours per night do you sleep? __________** hours
6. **How have your eating habits changed over the past year?**☐ Eating much more *(go to question 32)*
   ☐ Eating more *(go to question 32)*
   ☐ No change *(go to question 32)*
   ☐ Eating less
   ☐ Eating much less
7. **Why have you reduced your food intake in the past year?** _________________________
8. **Have you had a significant/serious illness in the past year?**☐ Yes, please specify the illness(es) you have had __________________________
   ☐ No
9. **Have you changed coach in the past few months?**☐ Yes
   ☐ No
10. **Have you changed club/team in the past few months?**☐ Yes
    ☐ No

# MENTAL HEALTH

1. Have you ever felt mentally ill during a period (daily for at least 2 weeks) so that you experienced significant difficulties in functioning normally in everyday life and sports

☐ Yes

☐ No

1. Have you any diseases that you take medication for regulary? (i.e. asthma, migrain etc.)

☐ Yes, namely ______________________________________________________

☐ No

Please indicate how well the statements under match how you have experienced your situation over the past 2 weeks

1. I’ve been feeling optimistic about the future past 2 weeks

☐ Always

☐ Often

☐ Sometimes

☐ Seldom

☐ Never

1. I’ve been feeling useful past 2 weeks

☐ Always

☐ Often

☐ Sometimes

☐ Seldom

☐ Never

1. I’ve been feeling relaxed past 2 weeks

☐ Always

☐ Often

☐ Sometimes

☐ Seldom

☐ Never

1. I’ve been dealing with problems well past 2 weeks

☐ Always

☐ Often

☐ Sometimes

☐ Seldom

☐ Never

1. I’ve been thinking clearly past 2 weeks

☐ Always

☐ Often

☐ Sometimes

☐ Seldom

☐ Never

1. I’ve been feeling close to other people past 2 weeks

☐ Always

☐ Often

☐ Sometimes

☐ Seldom

☐ Never

1. I’ve been able to make up my own mind about things past 2 weeks

☐ Always

☐ Often

☐ Sometimes

☐ Seldom

☐ Never

**The weekly questionnaire**

**INJURY or PAIN**

1. How much pain have you experienced as it worst past week? *(Enter a number between 0-10, regardless if you have been injured or not)*


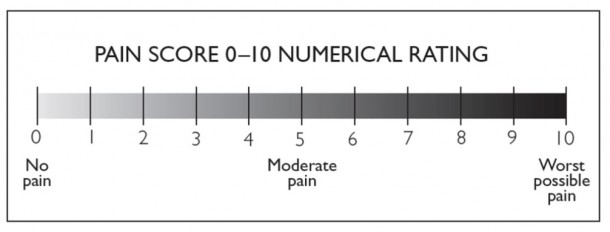


1. Have you had any difficulties participating in normal training and competition due to injury or pain during the **past 7 days?**

☐ Full participation without injury or pain *(go to question 7)*

☐ Full participation, but with injury or pain

☐ Reduced participation due to injury or pain

☐ Cannot participate due to injury or pain

1. To what extent have you reduced your training volume due to injury or pain past 7 days?

☐ No reduction

☐To a minor extent

☐ To a moderate extent

☐ To a major extent

☐ Cannot participate at all

1. Is it the same injury or pain as last week?

☐ Yes *(go to question 7)*

☐ No

1. Where on your body do you experience an injury or pain? NOTE! Right and left side are not important and that you can fill in multiple body localizations
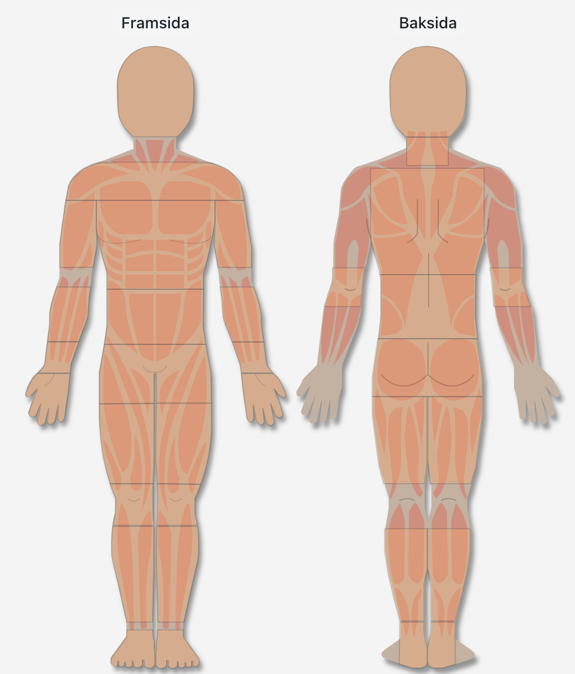


- Head or face, concussion or other head/face injury
- Neck
- Shoulder and clavicula
- Upper arm
- Elbow
- Forearm
- Wrist
- Hand or finger
- Chest or ribs
- Stomach
- Upper back or scapula
- Lower back
- Pelvis or buttock
- Hip or groin
- Anterior thigh
- Posterior thigh
- Knee
- Anterior lower leg
- Posterior lower leg
- Ankle or heel
- Foot or toes

1. How did the injury or pain occur?

☐ Sudden, without contact

☐ Sudden, with contact

☐ Gradually during training or match/competition

☐ Insidious and gradually without a specific/identifiable event/occasion

1. Have you been sick during past 7 days?

☐ Yes

☐ No

**TRAINING/MATCHPLAY/COMPETITION**

1. How many hours have you been training past 7 days? (*All kind of training*)

☐ 0-2 hours

☐ 3-5 hours

☐ 6-8 hours (approx. 1h/day)

☐ 9-11 hours (approx. 1.5h/day)

☐ 12-14 hours

☐ above 14 hours (more than 2h/day)

1. How many matches/competitions have you been participating in past 7 days?

☐ 0.5

☐ 1

☐ 1.5

☐ 2

☐ 2.5

☐ 3

☐ 4 or more

☐ I have not attended any match or competition

1. How would you rate your total week of training and match/competitions? *Choose a number between 0-10 based on the explanation below*

| 0 | Rest |
| --- | --- |
| 1 | Very easy |
| 2 | Easy |
| 3 | Moderate |
| 4 | Somewhat hard |
| 5 | Hard |
| 6 | Pretty hard |
| 7 | Very hard |
| 8 | Extremely hard |
| 9 | Almost maximal |
| 10 | Maximal |

**PSYCHOLOGICAL LOAD**

1. Please rate how you experienced your freshness/fatigue past 7 days?

☐Very fresh

☐ Somewhat fresh

☐ Normal

☐ More fatigue than usual

☐ Always fatigue

1. How was your sleeping quality past 7 days?

☐ Very good

☐ Good

☐ Hard to fall asleep

☐ Worried sleep (a lot awake)

☐ Sleeplessness/impossible to sleep

1. How was your stress level past 7 days?

☐ Very relaxed

☐ Relaxed

☐ Normal

☐ Stressed

☐ Very stressed

1. How do you experience you being mentally recovered past 7 days?

☐ Optimal/very good

☐ Good

☐ Moderate

☐ Bad

☐ Very bad/weak
